# Observation of an exotic insulator to insulator transition upon electron doping the Mott insulator CeMnAsO

**DOI:** 10.1038/s41467-023-42858-3

**Published:** 2023-11-03

**Authors:** E. J. Wildman, G. B. Lawrence, A. Walsh, K. Morita, S. Simpson, C. Ritter, G. B. G. Stenning, A. M. Arevalo-Lopez, A. C. Mclaughlin

**Affiliations:** 1 The Chemistry Department, University of Aberdeen, Meston Walk, Aberdeen, AB24 3UE UK; 2 Department of Materials, Imperial College London, London, SW7 2AZ UK; 3 Institut Laue Langevin, 71 Avenue des Martyrs, BP 156, F-38042 Grenoble, Cedex 9 France; 4 ISIS Experimental Operations Division, Rutherford Appleton Laboratory, Harwell Science and Innovation Campus, Didcot, OX11 0QX UK; 5 Université de Lille, CNRS, Centrale Lille, ENSCL, Université d’Artois, UMR 8181-UCCS-Unité de Catalyse et Chimie du Solide, F-59000 Lille, France

**Keywords:** Electronic properties and materials, Quantum physics

## Abstract

A promising route to discover exotic electronic states in correlated electron systems is to vary the hole or electron doping away from a Mott insulating state. Important examples include quantum criticality and high-temperature superconductivity in cuprates. Here, we report the surprising discovery of a quantum insulating state upon electron doping the Mott insulator CeMnAsO, which emerges below a distinct critical transition temperature, *T*_II_. The insulator-insulator transition is accompanied by a significant reduction in electron mobility as well as a colossal Seebeck effect and slow dynamics due to decoupling of the electrons from the lattice phonons. The origin of the transition is tentatively interpreted in terms of many-body localization, which has not been observed previously in a solid-state material.

## Abstract (web summary)

Doping a Mott insulator can lead to novel electronic states. Wildman et al. observe a novel quantum insulating state in electron-doped Mott insulator CeMnAsO and propose a tentative interpretation in terms of many-body localization, which has not been observed in a solid-state material.

## Introduction

**Fig. 1:**
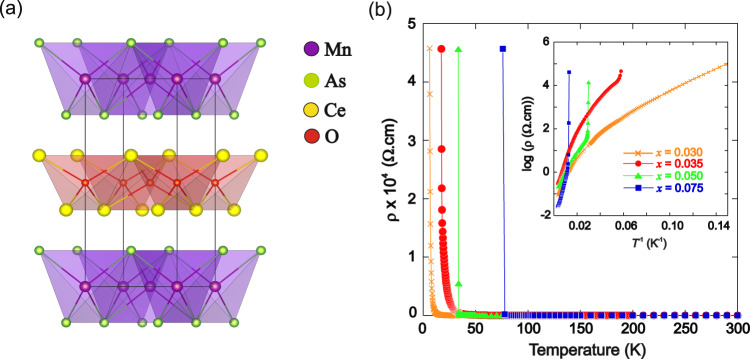
The crystal structure and DC electronic properties of CeMnAsO_1-*x*_F_*x*_. **a** The tetragonal unit cell of CeMnAsO_1-*x*_F_*x*_, which crystallises in the *P*4*/nmm* space group. Insulating CeO_1-*x*_F_*x*_ layers are situated between tetrahedral layers of MnAs. **b** The temperature-dependent resistivity (*ρ*) of CeMnAsO_1-*x*_F_*x*_, showing an insulator-insulator transition for *x* > 0.03. The data are normalised so that the transitions are apparent. The inset shows the variation of log(resistivity) versus inverse temperature where the insulator-insulator transition is observed for *x* = 0.035, 0.050 and 0.075.

The electronic properties of quasi-two-dimensional *LnM*AsO (Fig. 1a; *Ln* = lanthanide, *M* = transition metal) pnictides can be transformed with a small degree of electron doping. High-temperature superconductivity is observed with just 5% F doping in LaFeAsO_0.95_F_0.05_^1^. In stark contrast to the *Ln*FeAsO oxypnictides, which have a metallic ground state, *Ln*Mn*Pn*O (*Ln* = lanthanide; *Pn* = As, P) are Mott insulators^2–4^. These phases have drawn interest due to their magnetoresistance (MR) properties. MR is defined as a change in resistivity upon the application of a magnetic field, H, such that MR = ((*ρ*_H_ − *ρ*_0_)/*ρ*_0_), where *ρ*_0_ and *ρ*_H_ are the resistivities in zero and applied field, respectively. Magnetoresistant materials find technological application in sensing and spintronics devices. Discovering and employing new magnetoresistant materials with enhanced properties thus represents an ongoing goal. For NdMnAsO, exchanging just 5% of the oxygen site for fluorine induces colossal magnetoresistance (CMR) such that MR_9T_ (3 K) = −95%^5,6^. In contrast, PrMnAsO_0.95_F_0.05_ exhibits a structural phase transition at 34 K, driven by the Pr 4f electrons degrees of freedom which also results in a sizeable *MR*_7T_ (12 K) = −13.4%^7^.

In order to further investigate the electronic properties of Mn oxypnictides, we have now synthesised CeMnAsO_1-*x*_F_*x*_ (*x* = 0–0.075). Here we show that upon electron doping CeMnAsO_1-*x*_F_*x*_, a transition from the Mott insulating phase to a distinct quantum insulating phase is observed below a critical temperature *T*_II_. The transition has no thermodynamic signature and is a purely dynamical quantum criticality.

## Results and discussion

**Fig. 2:**
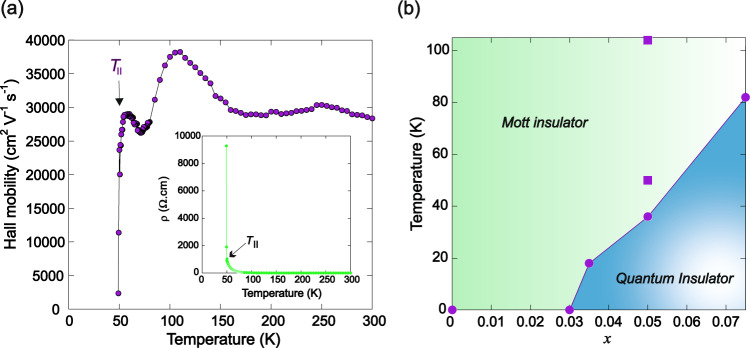
Hall mobility and resistivity of Ce_0.97_MnAsO_0.95_F_0.05_ and electronic phase diagram of Ce_*y*_MnAsO_1-*x*_F_*x*_. **a** The temperature dependence of the Hall mobility of Ce_0.97_MnAsO_0.95_F_0.05_ (defined as |*R*_H_(T)|/*ρ*(T), where *R*_H_(T) is the Hall coefficient recorded in H = 5000 Oe and *ρ* is the resistivity). A small drop in the Hall mobility is detected at the transition to variable range hopping (100 K) followed by a dramatic reduction in the mobility of the electrons at *T*_II_. The resistivity is shown in the inset and becomes too high to measure below 48.5 K. **b** The phase diagram shows the boundary of the Mott insulating phase and the quantum insulating phase for *x* = 0.00–0.075. The filled circles show *T*_II_ for Ce stoichiometric phases and the filled squares shows *T*_II_ for Ce_y_MnAsO_0.95_F_0.05_ (*y* = 0.96 and 0.97).

CeMnAsO_1-*x*_F_*x*_ (*x* = 0–0.075) phases have been synthesised and the chemical characterisation is described in the Supplementary Information (Supplementary Figs. 1 and 2 and Supplementary Tables 1–3). The parent compound, CeMnAsO, is a Mott insulator (*ρ*_290 K_ = 1.76 × 10^4^ Ω cm) as previously reported^3,8^. Figure 1b and Supplementary Fig. 3 show the temperature variation of the resistivity, *ρ*, for CeMnAsO_1-*x*_F_*x*_. For all *x*, between ∼300 ∼ 100 K the electron transport is dominated by thermally activated charge carriers across a band gap (*E*_g_). Below ∼100 K a transition from Arrhenius behaviour to Mott three-dimensional variable-range hopping (VRH) is observed in CeMnAsO_1-*x*_F_*x*_ for *x* > 0 so that the electrons are localised (Supplementary Fig. 4 and Supplementary Table 4). Below this temperature, transport can be described by phonon-assisted tunnelling of electrons between localised states. Surprisingly, upon further cooling, an insulator-insulator transition is observed for *x* > 0.03 where the resistivity increases by more than two orders of magnitude over a narrow temperature interval below a distinct temperature, *T*_II_ (Fig. 1b). The samples rapidly become too resistive to measure for temperatures <*T*_II_. The insulator-insulator transition is also apparent from Hall resistivity measurements, where a sharp and significant drop in the Hall mobility occurs at *T*_II_ (Fig. 2a and Supplementary Fig. 5). This significant reduction in electron mobility further triggers a colossal Seebeck effect at *T*_II_ (Supplementary Fig. 6)^9^. Such a quantum insulating phase emerging from a Mott insulator is surprising. The transition is also distinct from Efros-Shklovskii (ES) variable range hopping where a subtle electronic transition is observed as a soft Coulomb gap pinned at the Fermi level opens up at low temperature as a result of enhanced Coulomb correlations, as reported for NdMnAsO_1-*x*_F_*x*_^5^ (Supplementary Fig. 7).

The electronic phase diagram of CeMnAsO_1-*x*_F_*x*_ is shown in Fig. 2b. The transition temperature from the Mott insulator to the quantum insulating phase can be tuned by varying *x*. It is also possible to control *T*_II_ by manipulating the composition, as the transition appears to be highly sensitive to chemical non-stoichiometry (Supplementary Table 4). Two Ce-deficient samples exhibit *T*_II_s of 104 K and 50 K. The Ce occupancies were determined from Rietveld refinement using high-resolution X-ray diffraction data and the stoichiometries refined to Ce_0.96_MnAsO_0.95_F_0.05_ and Ce_0.97_MnAsO_0.95_F_0.05_ respectively. Although it is not possible to fully elucidate the exact Ce and F stoichiometry through X-ray diffraction and/or EDX, the results strongly suggest that cerium deficiency significantly enhances *T*_II_ (Supplementary Figs. 8 and 9), and that further study of Ce deficient samples is warranted. For more details see the Supplementary Information.

Resistivity changes of several orders of magnitude, within a narrow temperature range, have attracted technological interest for a range of practical applications in functional devices. Notable examples include VO_2_, which exhibits a change of lattice structure and a Mott metal-insulator transition^10^, and magnetite, where the transition to insulating behaviour is driven by charge ordering involving three-site distortions called trimerons^11^. Such temperature-driven metal-insulator and insulator-insulator transitions are generally caused by subtle changes in the crystal lattice and/or magnetic structure. However, magnetic susceptibility measurements show *T*_II_ is not coupled to any magnetic transition (Supplementary Figs. 11 and 12). Variable temperature powder neutron diffraction data recorded for Ce_0.96_MnAsO_0.95_F_0.05_ with *T*_II_ = 104 K confirm there is no change in the nuclear or magnetic structure at *T*_II_ (Supplementary Figs. 12–14 and Supplementary Tables 5 and 6). Heat capacity measurements on the same sample also show no evidence of any transition at *T*_II_ (Supplementary Fig. 15). Furthermore, high-resolution neutron diffraction data recorded for CeMnAsO_1-*x*_F_*x*_ (*x* = 0.00, 0.035, 0.050 and 0.075) at 300 K and 10 K also show that there is no change in crystal symmetry upon cooling (Supplementary Figs. 16 and 17). These results rule out other possible mechanisms for the insulator-insulator transition such as charge ordering, a spin state transition, a magnetic or structural transition, or orbital ordering, all of which would show a signature in either the magnetic or neutron diffraction or heat capacity data. The presence of an insulator-insulator transition with no change in the crystal or magnetic structure is highly unusual and suggests a more exotic phenomenon drives the significant increase in resistivity at *T*_II_.

Electron doping of several Mott-insulating Mn oxypnictides (*Ln*MnAsO_1-*x*_*X*_*x*_) (*Ln* = La, Pr, Nd, Sm; *X* = F^−^, H^−^ or a vacancy) has been performed previously^2,5,7,12^. The transition from insulating to metallic behaviour is normally observed at *x* ≈ 0.2. The conductivity observed in materials with *x* < 0.2 comes from charge carriers in the gap states that remain localised so that variable range hopping of the electrons is observed below ∼100 K. Typically reported values of *T*_0_ (*T*_0_ quantifies the degree of electronic disorder) are 4–7 × 10^5^ K for *Ln*MnAsO_1-*x*_F_*x*_ (*x* = 0.05–0.08) (*Ln* = Nd, Pr)^5,7^. For CeMnAsO_1-*x*_F_*x*_ and Ce_*y*_MnAsO_0.95_F_0.05_, *T*_0_ ranges from 1.88 × 10^6^ K–1.89 × 10^7^ K (Supplementary Table 4), which suggests that a much greater degree of Anderson electronic disorder is present in this series (see Supplementary Information for more details on the electronic properties).

**Fig. 3:**
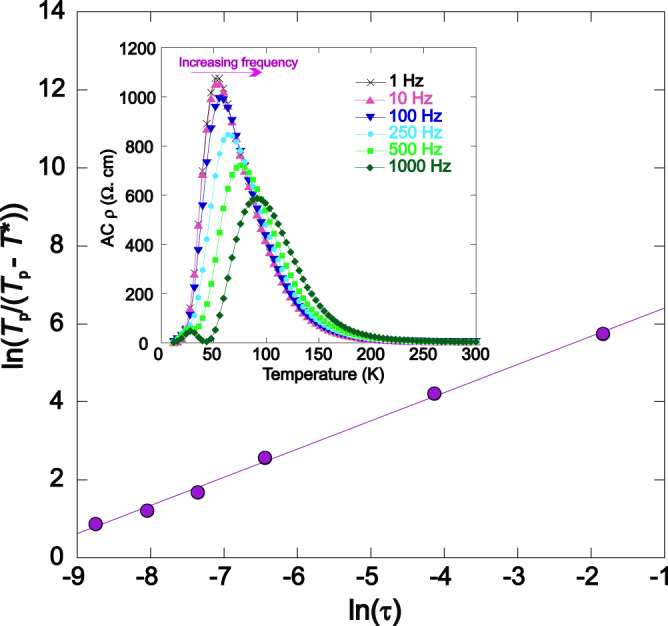
The AC transport of Ce_0.97_MnAsO_0.95_F_0.05_ with *T*_II_ = 50 K. The inset shows the temperature variation of the dissipative part of the AC resistivity of Ce_0.97_MnAsO_0.95_F_0.05_ at selected frequencies between 1 Hz and 1000 Hz. A peak is observed at *T*_II_ and moves to higher temperature with increasing frequency. The frequency dependence of the peak obeys the power law for Ce_0.97_MnAsO_0.95_F_0.05_. The power law states: ${\tau}={{\tau}}^{*}{\left[\frac{{T}_{{{{{{\rm{p}}}}}}}}{({T}_{{{{{{\rm{p}}}}}}}-{T}^{*})}\right]}^{ZV}$ where *τ* is a characteristic time describing the dynamical fluctuation time scale, τ* is the relaxation time constant, *T*_*p*_ is the temperature of the peak maximum temperature at a given frequency, T^*^ is the critical temperature, *z* is the dynamical critical exponent and *v* is the correlation length exponent.

The lack of a peak in the heat capacity at *T*_II_ (Supplementary Fig. 15) suggests that the transition has no thermodynamic signature and is a purely dynamical quantum criticality. To further explore the origin of the transition, variable-temperature and variable-frequency AC transport measurements were recorded for Ce_0.97_MnAsO_0.95_F_0.05_, Ce_0.96_MnAsO_0.95_F_0.05_ and CeMnAsO_0.0965_F_0.035_. The dissipative part of the AC conductivity is shown in Fig. 3 and Supplementary Figs. 18 and 19, respectively. A broad peak is observed at T* which is slightly higher than *T*_II_ (T* = 53 K for Ce_0.97_MnAsO_0.95_F_0.05_ (*T*_II_ = 50 K), 112 K for Ce_0.96_MnAsO_0.95_F_0.05_ (*T*_II_ = 104 K) and 26 K for CeMnAsO_0.0965_F_0.035_ (*T*_II_ = 18 K)). The peak exhibits a clear frequency response, typical of a dynamical system, and is reminiscent of the variable-frequency properties of other materials which exhibit dynamic phenomena such as spin and dipole glasses. The frequency dependence of the peak temperature (T*) is well modelled by the power law (Fig. 3 and Supplementary Fig. 18) with relaxation time constants of 4.2(2) × 10^−5^ s, 7.2(3) × 10^−5^ s and 3.2(2) × 10^−5^ s and *zv* = 1.4(1), 1.1(2) and 1.3(2) for Ce_0.97_MnAsO_0.95_F_0.05_, Ce_0.96_MnAsO_0.95_F_0.05_ and CeMnAsO_0.0965_F_0.035_ respectively. The fit to the power law demonstrates that there is a critical slowing of dynamics at T^*^.

The electronic behaviour at *T*_II_ is distinct from an electron glass. The transition reported here is sensitive to the applied AC frequency so that a very low frequency is required to modify the transition (Fig. 3 and Supplementary Figs. 18 and 19). Exotic electron glasses such as the marginal Fermi glass observed in phosphorus-doped silicon (Si:P)^13^ and the electron glass indium oxide^14^ only show anomalous behaviour at much higher frequencies (MHz–THz). In these materials, it has been proposed the glassy behaviour only emerges when the applied AC frequency is similar to the phonon-electron relaxation time, as the presence of an AC frequency similar to the phonon relaxation time disrupts the ability for the electrons to interact with the phonons. For the Ce_*y*_MnAsO_1-*x*_F_*x*_ phases, slow dynamics are observed at much lower frequencies of ~1 Hz, which indicates the electrons are much more poorly coupled to thermal reservoirs such as phonons than they are in established electron glasses. The relaxation times we extracted from the AC resistivity data (~10^–5^ s) are also significantly slower than the typical phonon or electron-phonon relaxation times. Variable frequency AC transport measurements such as those reported here are therefore ideal to distinguish the quantum insulating phase observed in Ce_*y*_MnAsO_1-*x*_F_*x*_ from a glassy system.

The results from the AC transport measurements would suggest that the observed slowing of dynamics in Ce_*y*_MnAsO_1-*x*_F_*x*_ is due to a gradual decoupling between the electrons and phonons. This is highly unusual and shows that there is a transition from an open to a closed system upon cooling. It has previously been reported that it is possible for electrons and phonons to decouple, if the interacting electrons are not equilibrated with the crystal lattice, which can arise due to electron overheating in strongly disordered systems^15,16^. Further research is needed to fully elucidate the mechanism behind the electron-phonon decoupling in Ce_*y*_MnAsO_1-*x*_F_*x*_.

**Fig. 4:**
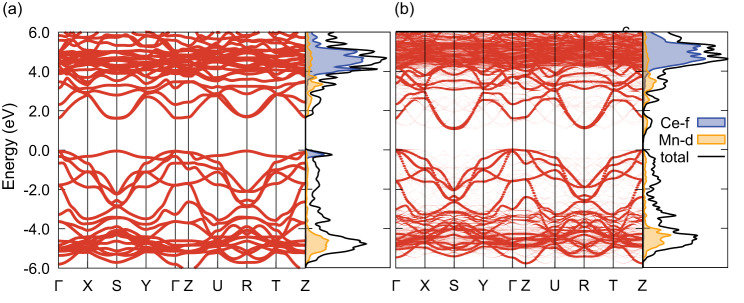
Calculated electronic properties of CeMnAsO and CeMnAsO_0.94_F_0.06_. **a** The electronic band structure and density of states of stoichiometric CeMnAsO. The partial density of states calculated with respect to Ce 4f and Mn 3d projections are shaded in blue and yellow, respectively. The energy was shifted so that 0.0 eV corresponds to the Fermi energy. **b** The unfolded electronic band structure and density of states of CeMnAsO_0.94_F_0.06_. The unfolding was done to match the reciprocal space path of the stoichiometric system. The energy was shifted so that 0.0 eV corresponds to the valence band maximum (Fermi energy at 2.0 eV). Both results are for the majority spin channel (the minority spin channel is shown in Supplementary Figs. 20 and 21).

To gain further insight into the origin of the insulator-insulator transition in Ce_*y*_MnAsO_1-*x*_F_*x*_ phases, density functional theory (DFT) calculations including non-local electron exchange were performed. The electronic band structures of CeMnAsO and CeMnAsO_0.94_F_0.06_ are presented in Fig. 4a, b, respectively. The narrow band at the valence band maximum in Fig. 4a, which originates from the Ce-4f state, is clearly absent in Fig. 4b. This suggests that, surprisingly, upon electron doping by substitution of F^−^ for O^2−^, the Ce 4f band is destabilised in the vicinity of the F^−^ anions and Ce^3+^ is oxidised to Ce^4+^. In CeMnAsO, Ce^3+^ has a formal 4f^1^ electronic configuration and upon electron doping the system reverts to a stable Ce^4+^ 4f^0^ configuration as found in CeO_2_. This suggests that in CeMnAsO, the 4f^0^ and 4f^1^ electronic configurations are competing with initial preference for the 4f^1^ state and that this subtle balance is broken by the substitution of F^−^. Charge neutrality is preserved as the excess electrons, upon oxidation of Ce^3+^ to Ce^4+^, move from the insulating CeO/F charge transfer layer to the As-Mn-As block as itinerant electrons.

The results from the quantum mechanical calculations are confirmed by neutron diffraction data recorded at 4 K for CeMnAsO_1-x_F_x_ which show a significant reduction in the ordered Ce moment with F^−^ doping, as would be expected upon oxidation of Ce^3+^ to Ce^4+^ (Supplementary Fig. 22). However, the reduction in the Ce moment upon increasing the F^−^ concentration from 0–0.05 is 16%, which would suggest that while there is some oxidation of Ce^3+^ to Ce^4+^, it is not uniform throughout the whole sample. It is therefore likely that domains of Ce^3+^ and Ce^4+^ coexist within the structure.

Chemical doping of Mott-insulators leads to chemical disorder which then results in electronic inhomogeneity and hence electronic disorder^17^. The Anderson-Hubbard model is used to describe doped transition metal oxides or oxypnictides such as Ce_*y*_MnAsO_1−*x*_F_*x*_, where the electronic properties arise due to doping-related disorder and strong local Coulomb potential^18^. The electronic disorder and inhomogeneity that arises upon doping Mott insulators can also lead to exotic states inside the Mott gap^19^. For Ce_*y*_MnAsO_1-*x*_F_*x*_, the electronic disorder is amplified as it is likely that the phases will contain insulating regions of localised correlated electrons and metallic regions of itinerant electrons from the 5% F^−^ doping and subsequent oxidation of Ce^3+^ in the vicinity of the F^-^ ion. Such mixed electronic states can lead to exotic properties and is most likely the origin of the quantum insulating state in Ce_*y*_MnAsO_1-*x*_F_*x*_. It is surprising that such a large increase in *T*_II_ is observed upon decreasing the Ce stoichiometry from *y* = 1–0.96 (*T*_II_ increases from 34 K–104 K). The presence of Ce non-stoichiometry could further enhance the oxidation of Ce^3+^ to Ce^4+^ and hence increase the electronic disorder which would explain why there is such a significant increase in *T*_II_ with very small changes in Ce stoichiometry.

Given that *T*_II_ is an entirely dynamical transition, observed in an electronically disordered phase, and that AC transport measurements show that there is a change from an open to a closed system upon cooling, a potential origin of the localisation transition observed in Ce_*y*_MnAsO_1-*x*_F_*x*_ at *T*_II_ is many body localisation (MBL). MBL has previously been described by theoretical and numerical studies of isolated quantum systems^20–22^, and transiently probed with ultra-cold atoms within isolated small systems on finite times^23,24^. MBL arises within a disordered system of interacting particles when quantum interference effects restrict transport to prevent the system from attaining thermal equilibrium under its own internal dynamics^21,22^. The transition to the MBL state is also characterised by singularities only in dynamical quantities^25^, so it does not require a breaking of symmetry. MBL systems have been proposed to serve as a form of quantum memory, in which information may be preserved in local observables across arbitrarily long timescales^21,26^. Furthermore, MBL systems may also be exploited as a promising platform to access alternative exotic quantum phases^26^, including the recently discovered discrete time crystal^27^.

Signatures of MBL have previously been detected in a handful of materials. Thin films of amorphous indium oxide exhibit vanishing conductivity in proximity to a magnetic-field-induced superconductor-insulator transition, which was attributed to the onset of a finite-temperature insulating state^28^. However, this was not accompanied by the characteristic slowing of dynamics that should precede the emergence of MBL^29^, so it most likely has an alternative physical origin. Nuclear magnetic resonance experiments on fluorapatite single crystals have revealed that the correlation length of the quasi-1D chains of spin-1/2 ^19^F nuclei exhibits a similar evolution with time to the expected growth of entanglement entropy in the MBL regime^30^. However, while this resembles the theoretically expected response for MBL, further experimental signatures have yet to be reported in this material. AC magnetic susceptibility measurements on a single crystal of the disordered cluster magnet LiHo_0.045_Y_0.955_F_4_, revealed that interactions between spin clusters and residual free spins within the wider system can be tuned by a magnetic field such that magnetic dipole excitations can become localised^31^. This suggests that the residual spins can no longer act as a bath with respect to the spin clusters, and the system appears to become dissipationless. In this sense, LiHo_0.045_Y_0.955_F_4_ shows promise for studying how fragile non-equilibrium phenomena interact with a thermal reservoir, but ultimately no other behaviour consistent with MBL has been detected in this material.

The transition to the quantum insulating phase in Ce_*y*_MnAsO_1-*x*_F_*x*_ (*x* > 0.03) does not arise due to a change in the crystal or magnetic structure. Upon cooling, a critical slowing of dynamics is observed as the electrons and phonons decouple. An insulator-insulator transition is then observed at *T*_II_. This transition is not accompanied by a thermodynamic signature and below *T*_II_ there is vanishing conductivity, a rapid change in the charge mobility and a colossal Seebeck effect as the electrons become localised. The Ce_*y*_MnAsO_1-*x*_F_*x*_ phases are electronically inhomogeneous and likely contain insulating regions of localised correlated electrons and metallic regions of itinerant electrons. Given the dynamical nature of the electron localisation transition at *T*_II_, we hypothesise that the transition could be a result of MBL. However, the electronic structure of Ce_y_MnAsO_1-x_F_x_ is pseudo two-dimensional and it is not yet clear whether MBL is possible in two-dimensional electronic phases^32–36^. Further experimental and theoretical studies are warranted to corroborate MBL in Ce_*y*_MnAsO_1-*x*_F_*x*_. If confirmed, the discovery of this novel MBL phase emerging from a Mott insulator will inspire further understanding of non-equilibrium quantum statistical mechanics and could revolutionise next-generation technological applications involving quantum computing. Further studies of the electronic phase diagram of Ce_*y*_MnAsO_1-*x*_F_*x*_ are also clearly warranted to explore the boundaries between existing quantum and classical models.

## Methods

### Synthesis

Polycrystalline samples of CeMnAsO_1-*x*_F_*x*_ (*x* = 0.00, 0.030, 0.035, 0.050 and 0.075) and Ce_*y*_MnAsO_0.95_F_0.05_ were synthesised via a two-step solid-state reaction method. Initially, the CeAs precursor was obtained by the reaction of Ce pieces (Aldrich 99.9%) and As (Alfa Aesar 99.999%) at 980 °C for 33 h in an evacuated, sealed quartz tube. The resulting precursor was then reacted with stoichiometric amounts of MnO_2_, Mn and MnF_2_ (Aldrich 99.99%), all powders were ground in an inert atmosphere and pressed into pellets of 10 mm diameter. The pellets were placed into a Ta crucible and sintered at 1150 °C for 48 h, again in a quartz tube sealed under vacuum.

### Diffraction

Room temperature powder X-ray diffraction patterns suitable for Rietveld refinement were recorded on a PANalytical Empyrean diffractometer using a Cu K*α*1 source (*λ* = 1.54059 Å) between 10 < 2*θ* < 120° with a step size of 0.013°. Powder neutron diffraction patterns were recorded on the high intensity diffractometer D20 at the Institute Laue Langevin (ILL, Grenoble, France) with a wavelength of 2.4188 Å. A 1 g sample of Ce_0.96_MnAsO_0.95_F_0.05_ was inserted into an 8 mm vanadium can contained in a cryofurnace and data were recorded between 5 and 400 K, with a collection time of ~20 min at each temperature. Data were also recorded on the high-resolution, two-axis diffractometer, D2B between 3.5 and 290 K in an 8 mm vanadium can with a collection time of ~3 h at each temperature.

Rietveld refinements^37^ of the structural model were carried out using the GSAS program^38^. The scale factor, background (shifted Chebyschev polynomial function), pseudo-Voigt profile function, lattice parameters, atomic positions, zero shift and atomic displacement parameters were all refined. The atomic displacement *U*-factors were refined isotropically.

### EDS analysis

EDS analysis was performed using a field emission gun Carl Zeiss Gemini SEM 300 equipped with an AZtec Energy EDS analysis system with an XMax 80 detector and an AZtecHKL EBSD analysis system with a Nordlys Nano EBSD camera (Oxford Instruments Ltd.). Energy dispersive spectroscopy (EDS) analysis was performed on CeMnAsO_1-*x*_F_*x*_ (*x* = 0, 0.035, 0.05 and 0.075) and Ce_0.97_MnAsO_0.95_F_0.05_. For each sample, data were collected on 15 randomly selected crystallites.

### Physical property measurements

The temperature dependence of the DC electrical resistance, Seebeck coefficient and Hall resistivity of the Ce_*y*_MnAsO_1-*x*_F_*x*_ phases were recorded using a Quantum Design physical property measurement system (PPMS) between 4 and 300 K. The measurements were performed with both the 2-probe and 4-probe methods to ensure the transitions were not a result of instrumental error. From ∼20 K above *T*_II_, data were also recorded every 0.25 K in order to obtain high quality and reliable data around the transition and to fully map out the transition. The temperature and frequency dependence of the AC transport of Ce_0.96_MnAsO_0.95_F_0.05_, Ce_0.97_MnAsO_0.95_F_0.05_ and CeMnAsO_0.0965_F_0.035_ were also recorded between 4 K and 300 K on the PPMS with selected frequencies between 1 Hz and 1000 Hz. Magnetisation measurements of all samples were recorded on a Quantum Design SQUID Magnetometer in an applied field of 100 Oe after zero field cooling (ZFC) and field cooling (FC) at temperatures between 2–400 K.

### First-principles calculations

The electronic structure was calculated using plane-wave density functional theory within projector-augmented wave scheme as implemented in VASP. A cut-off energy of 600 eV and reciprocal space sampling of 6 × 6 × 3 and 3 × 3 × 3 were used for CeMnAsO and CeMnAsO_0.94_F_0.06_, respectively^39–41^. For CeMnAsO_0.94_F_0.06_, the supercell size was expanded into a size of 3 × 3 × 1 (18 formula units) and one O was replaced by F. For all the calculations, the HSE06 exchange-correlation functional within collinear spin representation was used^42,43^. The lattice constants were taken from the experimental structural refinement and the internal atomic coordinates were fully relaxed. To compare the band structure between CeMnAsO and CeMnAsO_0.94_F_0.06_, which have a different cell size, the band structure in CeMnAsO_0.94_F_0.06_ was unfolded onto the Brillouin zone path in CeMnAsO using the *VASPBandUnfolding* package^44^ .The density of states was analysed and plotted with the *sumo* package^45^.

### Supplementary information

Supplementary Information

Peer Review File

## Data Availability

The computational input and output files have been uploaded to the Zenodo Repository and are available from https://zenodo.org/record/8403227. Neutron diffraction data are available from the ILL at 10.5291/ILL-DATA.5-23-707. Other data that support the findings of this study are available from the corresponding author upon request.
