# Supplementary material for: Observation of an exotic insulator to insulator transition upon electron doping the Mott insulator CeMnAsO
Source: Nat Commun. 2023 Nov 3;14:7037. doi: 10.1038/s41467-023-42858-3 (PMC10624918; doi:10.1038/s41467-023-42858-3)
Supplement: Supplementary file 1 — Supplementary Information [file 41467_2023_42858_MOESM1_ESM.pdf]

Supplementary information for

Observation of an Exotic Insulator to Insulator Transition upon Electron Doping the Mott Insulator CeMnAsO

E. J. Wildman [1], G. B. Lawrence [1], A. Walsh [2], K. Morita [2], S. Simpson [1], C. Ritter [3], G. B. G. Stenning [4], A. M. Arevalo-Lopez [5] and A. C. Mclaughlin [1*]

[1] The Chemistry Department, University of Aberdeen, Meston Walk, Aberdeen, AB24 3UE, Scotland.

[2] Department of Materials, Imperial College London, Exhibition Road, London SW7 2AZ, United Kingdom.

[3] Institut Laue Langevin, 71 Avenue des Martyrs, BP 156, F-38042 Grenoble Cedex 9, France.

[4] ISIS Experimental Operations Division, Rutherford Appleton Laboratory, Harwell Science and Innovation Campus, Didcot, OX11 0QX, UK.

[5] Université de Lille, CNRS, Centrale Lille, ENSCL, Université d'Artois, UMR 8181-UCCS-Unitéde Catalyse et Chimie du Solide, F-59000 Lille, France.

Supplementary Note 1. Sample Characterisation

EDS analysis shows the presence of uniformly distributed Ce, Mn, As, O and F so that the samples are compositionally homogeneous within the spatial resolution of SEM-EDS analysis. A typical EDS scan is shown in Supplementary Figure 1. The average atomic percentages calculated from the measurements are close to the theoretical values within instrument resolution and standard deviations as shown in Supplementary Table 1.

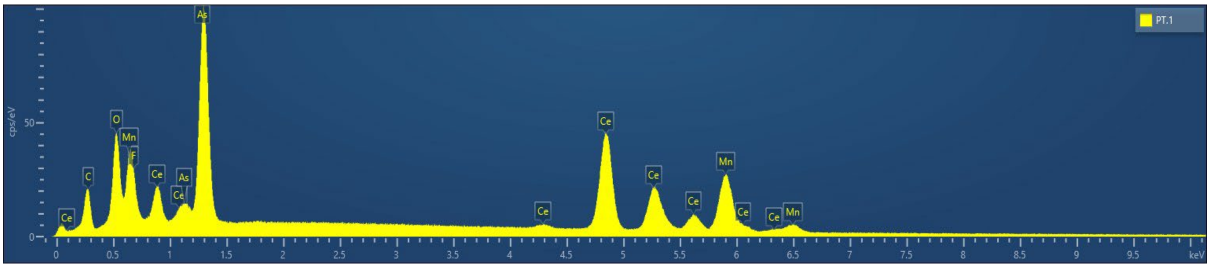

Supplementary Figure 1 Typical EDS scan for the sample $Ce_{0.97}MnAsO_{0.95}F_{0.05}$.

Supplementary Table 1 Results from EDS analysis on powders samples of $Ce_yMnAsO_{1-x}F_x$

	Mn%	As%	Ce%
**Theoretical**	25	25	25
**CeMnAsO**	23.94 (± 0.45)	24.30 (± 0.39)	25.28 (± 0.50)
**$CeMnAsO_{0.965}F_{0.035}$**	23.65 (± 0.42)	24.15 (± 0.37)	24.98 (± 0.49)
**$CeMnAsO_{0.95}F_{0.05}$**	23.71 (± 0.45)	24.46 (± 0.39)	24.90 (± 0.51)
**$Ce_{0.97}MnAsO_{0.95}F_{0.05}$**	23.58 (± 0.43)	24.23 (± 0.36)	24.931 (± 0.48)
**$CeMnAsO_{0.925}F_{0.075}$**	23.71 (± 0.41)	24.39 (± 0.35)	25.00 (± 0.47)

Within the accuracy of EDS measurements, the stoichiometry was shown to be 1:1:1 for all phases for Ce:Mn:As. The average compositions from the ratio between the atomic percentages are given in Supplementary Table 2.

Supplementary Table 2 Average compositions obtained from EDS analysis on powders samples of $Ce_yMnAsO_{1-x}F_x$.

	Mn	As	Ce
**CeMnAsO**	0.99	1	1.04
**$CeMnAsO_{0.965}F_{0.035}$**	0.98	1	1.03
**$CeMnAsO_{0.95}F_{0.05}$**	0.97	1	1.02
**$Ce_{0.97}MnAsO_{0.95}F_{0.05}$**	0.97	1	1.03
**$CeMnAsO_{0.925}F_{0.075}$**	0.97	1	1.03

It is not possible to determine the level of Ce non-stoichiometry through EDX analysis as the Ce vacancy concentration is too small to be determined accurately. We have synthesised over sixty $CeMnAsO_{0.95}F_{0.05}$ phases. After every synthesis, we have collected a 16-hour high resolution X-ray diffraction pattern and performed Rietveld refinement. DC resistivity results always show that samples that exhibit Ce non-stoichiometry from Rietveld refinement have higher transitions than stoichiometric $CeMnAsO_{0.95}F_{0.05}$ phases. It is also not possible to determine the O/F concentration through EDX analysis but the increase of the Ce-O/F bond length as described below demonstrates that nominal doping has occurred.

Laboratory powder X-ray diffraction patterns revealed that $CeMnAsO_{1-x}F_x$ phases with $x < 0.075$, $Ce_{0.96}MnAsO_{0.95}F_{0.05}$ and $Ce_{0.97}MnAsO_{0.95}F_{0.05}$ were single phase and could be indexed on the ZrCuSiAs-type tetragonal unit cell with the *P*4/*nmm* space group (Supplementary Fig. 2). A minor impurity phase, CeOF (~ 1.5%), is observed for $x = 0.075$, which would suggest the F^- concentration is less than 0.075. Attempts to chemically dope CeMnAsO further were unsuccessful, as secondary phases of CeF_2 and MnF_3 impurities began to emerge at $x > 0.075$. This would suggest that the doping limit is $x = 0.075$ for substitution of F^- for O^{2-}.

Rietveld refinements using X-ray diffraction data show that there is no trend in the unit cell parameters upon increasing *x* (Supplementary Table 3). However, the clear increase in Ce-O/F

with x shows that the nominal doping has been successful. In the superconducting $CeFeAsO_{1-x}F_x$ series, the same increase in the Ce-O/F bond length is observed with F^- doping. The effect of fluorine doping has been suggested to bring the CeO/F charge transfer layer closer to the conducting layer (As-Fe-As block), facilitating electron charge transfer[1]. The Rietveld refinement fits to the ZrCuSiAs-type tetragonal model are shown in Supplementary Figure 2 for all x.

Supplementary Table 3 Selected refined cell parameters and Ce-O/F bond lengths for the $CeMnAsO_{1-x}F_x$ series (x = 0 – 0.075) obtained from Rietveld fits against laboratory X-ray diffraction data recorded at room temperature.

x	**0.00**	**0.03**	**0.035**	**0.05**	**0.075**
***a* (Å)**	4.09010(1)	4.08756(1)	4.08834(1)	4.08812(1)	4.08873(1)
***c* (Å)**	8.96544(2)	8.95738(4)	8.95975(2)	8.95908(2)	8.96135(4)
**Volume ($Å^3$)**	149.982(1)	149.661(2)	149.758(1)	149.730(1)	149.813(1)
**Ce-O/F (Å)**	2.3613(1)	2.3619(2)	2.3624(2)	2.3626(2)	2.3627(2)

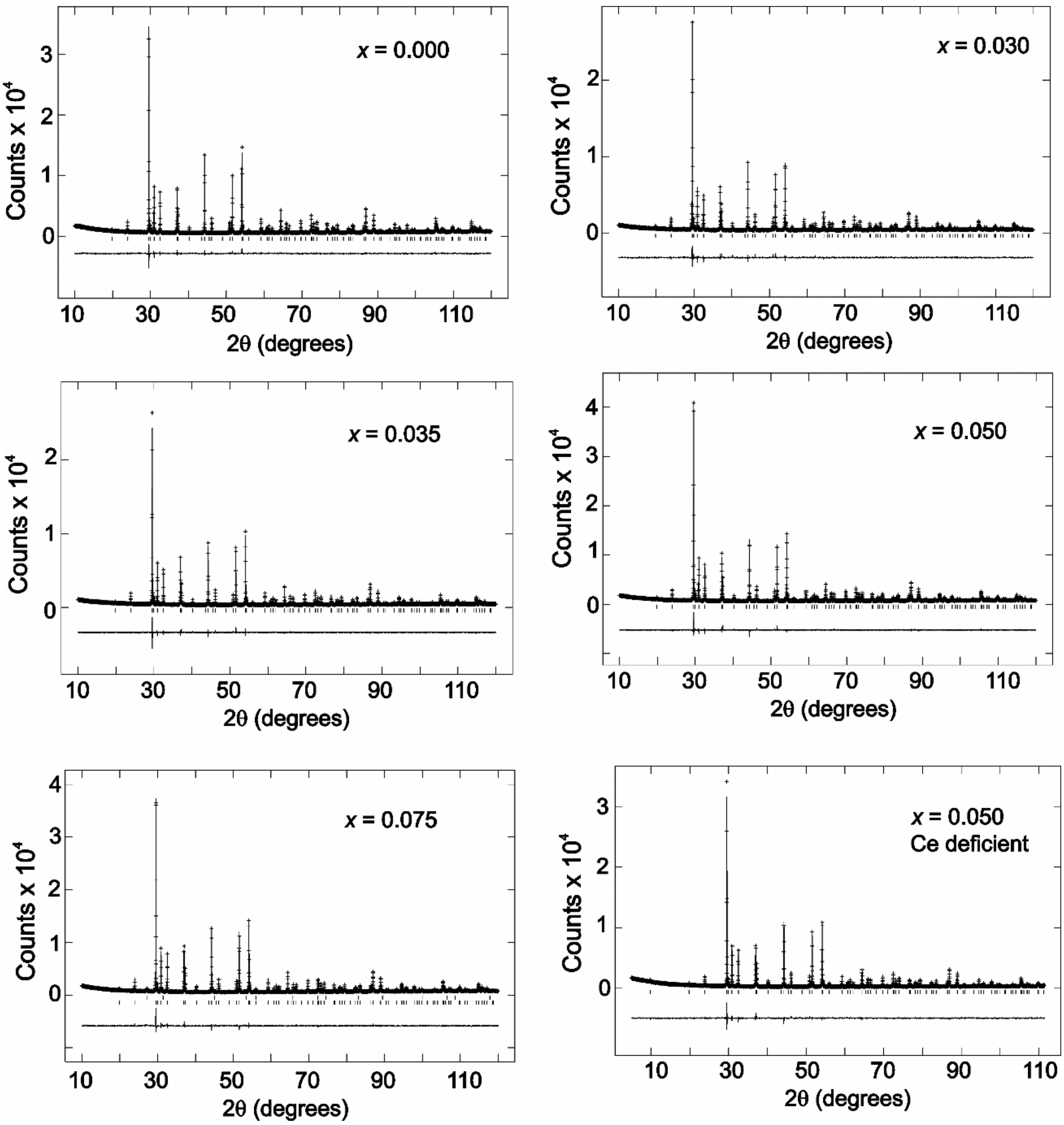

Supplementary Figure 2. Rietveld refinement fits from X-ray diffraction data of $CeMnAsO_{1-x}F_x$ and $Ce_{0.96}MnAsO_{0.95}F_{0.05}$ (Ce deficient). The secondary phase CeOF is included in the refinement fit for x = 0.075.

Supplementary Note 2. Electronic Properties

For all x, between ~ 300 ~ 100 K the electron transport is dominated by thermally activated charge carriers across a band gap (E_g) according to the relationship $\rho = \rho_0\exp(E_g/2kT)$, where ρ is the measured resistivity, k the Boltzmann constant and T is the temperature. The band gap (E_g) decreases from 0.32(2) eV for x = 0.0 to 0.08(2) eV for x = 0.075. This suggests that electron doping creates mid-gap states within the Mott-Hubbard gap so that the observed band gap reduces in magnitude with electron doping. Below ~ 100 K a transition from Arrhenius

behaviour to Mott three-dimensional variable range hopping (3D VRH) is observed for $x > 0$. Below this temperature, transport can be described by phonon-assisted tunnelling of electrons between localised states, so that ρ is defined as $\rho = \rho_0 \exp(T_0/T)^{0.25}$ and $T_0 = \lambda\alpha^3/kN(E_F)$, where T_0 describes the degree of electronic disorder, λ is a dimensionless constant, α^{-1} is equal to the localisation length and $N(E_F)$ is the density of localised states at E_F.

Supplementary Table 4. Variation of E_g, T_0 and T_{II} with x for the $CeMnAsO_{1-x}F_x$ solid solution and $Ce_xMnAsO_{0.95}F_{0.05}$ (Ce deficient). The band gap (E_g (calc)) from the DFT calculations is also shown for x = 0 and x = 0.06. The calculated band gap is a sizable overestimate, which is likely to originate from the limitation of DFT to treat the strongly correlated nature of the quasiparticle band gap in this material.

x	***E_g* (eV)**	**E_g(eV) (calc)**	**T_0 (K)**	**T_{II} (K)**
**0.000**	0.31(5)	1.68	-	-
**0.030**	0.05(3)		1.88(2) x 10^6	-
**0.035**	0.07(5)		6.73(3) x 10^6	18
**0.050**	0.04(2)		2.51(3) x 10^6	34
		1.04		
**0.075**	0.08(4)		- [(a)]	82
**0.050 ($Ce_{0.96}$)**	0.19(4)		- [(a)]	104
**0.050 ($Ce_{0.97}$)**	0.09(4)		1.89(3) x 10^7	50

(a) There was not a large enough temperature range to obtain reliable values of T_0 for x = 0.075 and 0.050 (Ce deficient) as a result of the higher T_{II}.

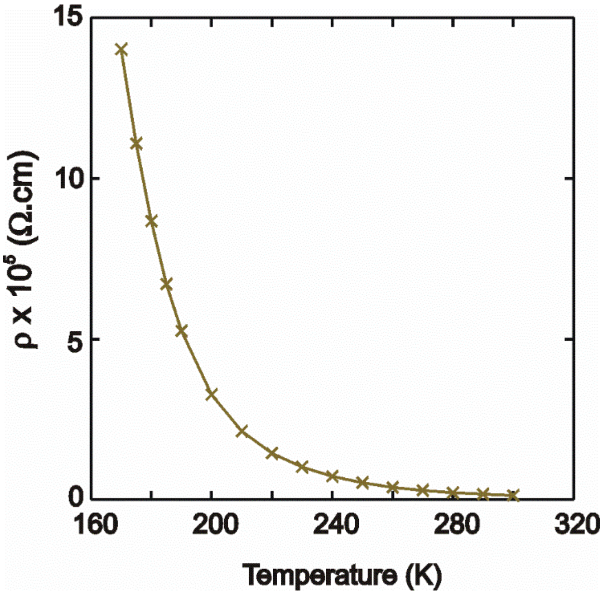

Supplementary Figure 3. Variable-temperature resistivity data for CeMnAsO. CeMnAsO is a Mott insulator and is too resistive to measure below 170 K.

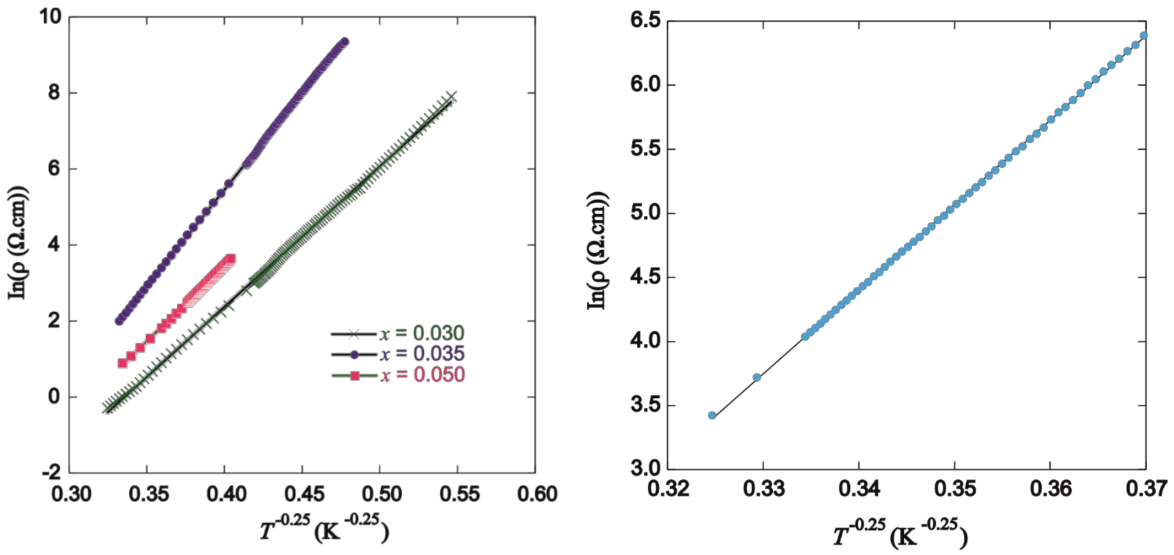

Supplementary Figure 4. The fits to the 3-dimensional variable range hopping (VRH) equation for $CeMnAsO_{1-x}F_x$ with x = 0.030, 0.035 and 0.050 (left) and $Ce_{0.97}MnAsO_{0.95}F_{0.05}$ (right).

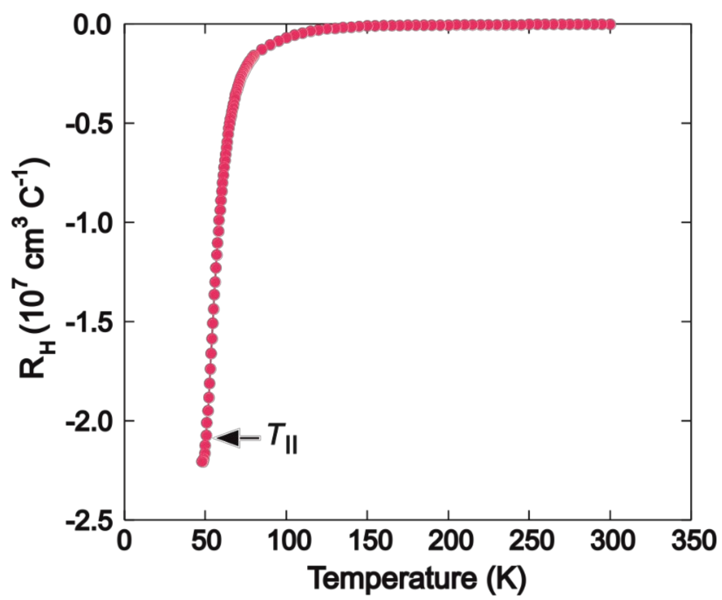

Supplementary Figure 5. The variation of the Hall coefficient with temperature for $Ce_{0.97}MnAsO_{0.95}F_{0.05}$. T_{II} for this sample is highlighted with an arrow.

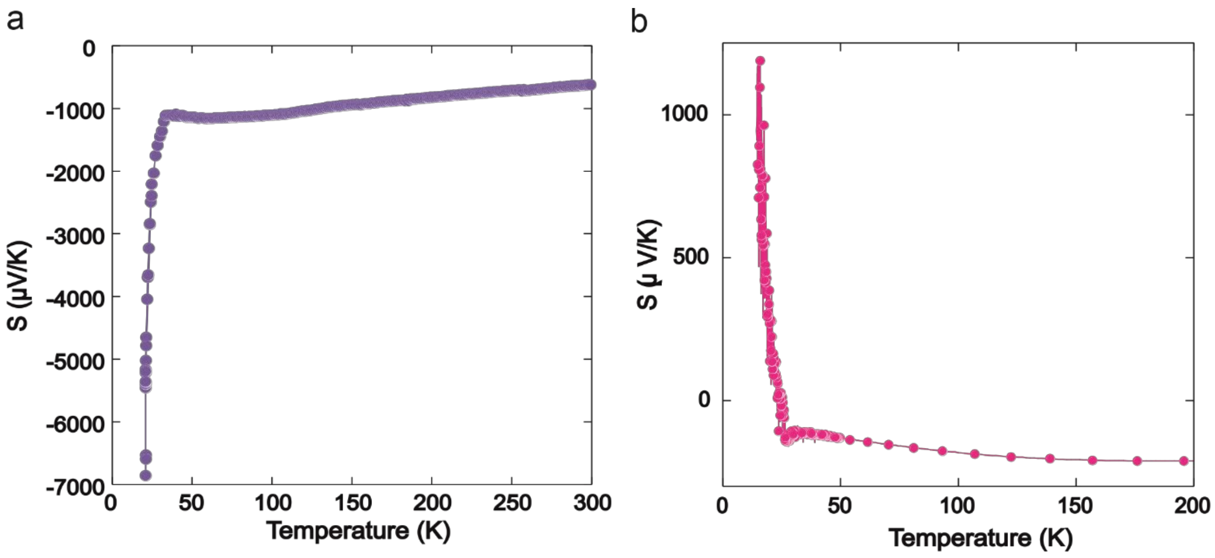

Supplementary Figure 6. The variation of the Seebeck coefficient with temperature for $CeMnAsO_{1-x}F_x$ samples with T_{II} equal to (a) 21 K where a colossal negative Seebeck effect is observed and (b) 18 K where a colossal positive Seebeck effect is observed. The nominal electron doping for both phases is $x = 0.035$ but the transitions are slightly different (21 K and 18 K which would suggest a higher electron doping for the sample with a T_{II} of 21 K). The Seebeck coefficient is sensitive to the position of the Fermi level. The change from positive to negative values most likely arises due to a small change in the electronic chemical potential as previously reported by Markov *et al.* [2]

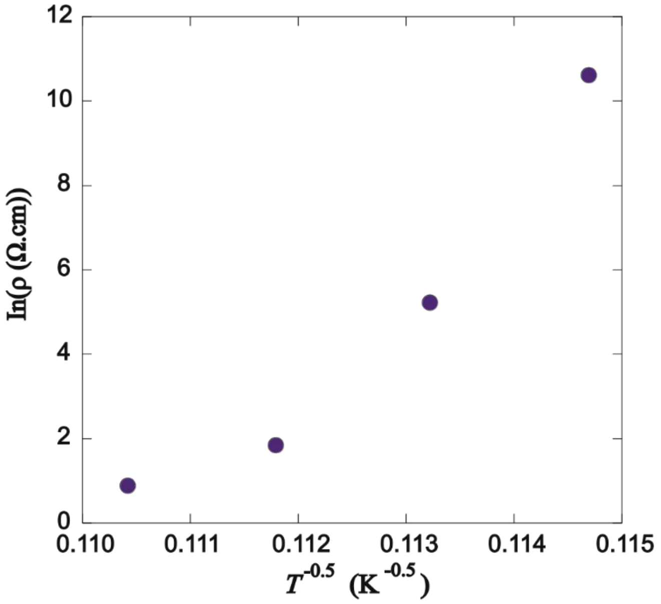

Supplementary Figure 7. The variation of ln(resistivity) versus $T^{-0.5}$ for $CeMnAsO_{0.925}F_{0.075}$ below 82 K showing that the transition is not a result of a transition to Efros Shklovskii (ES) variable range hopping below T_{II}.

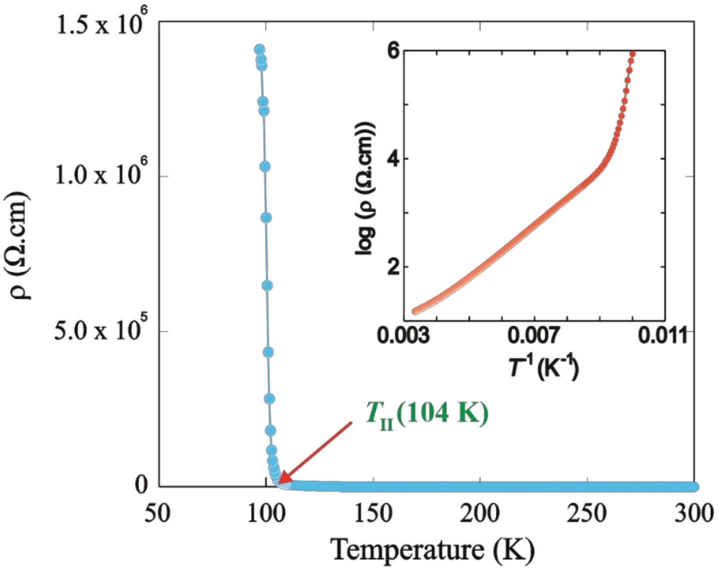

Supplementary Figure 8. The variation of the resistivity of $Ce_{0.96}MnAsO_{0.95}F_{0.05}$ with temperature, evidencing an insulator-insulator transition at 104 K. The inset shows the temperature dependence of the log resistivity where T_{II} is evident.

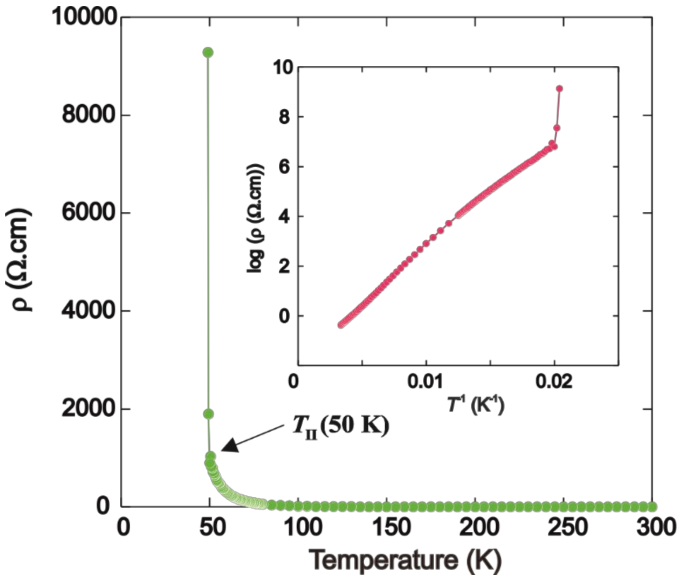

Supplementary Figure 9. The variation of the resistivity of $Ce_{0.97}MnAsO_{0.95}F_{0.05}$ with temperature, evidencing an insulator-insulator transition at 50 K. The inset shows the temperature dependence of the log resistivity.

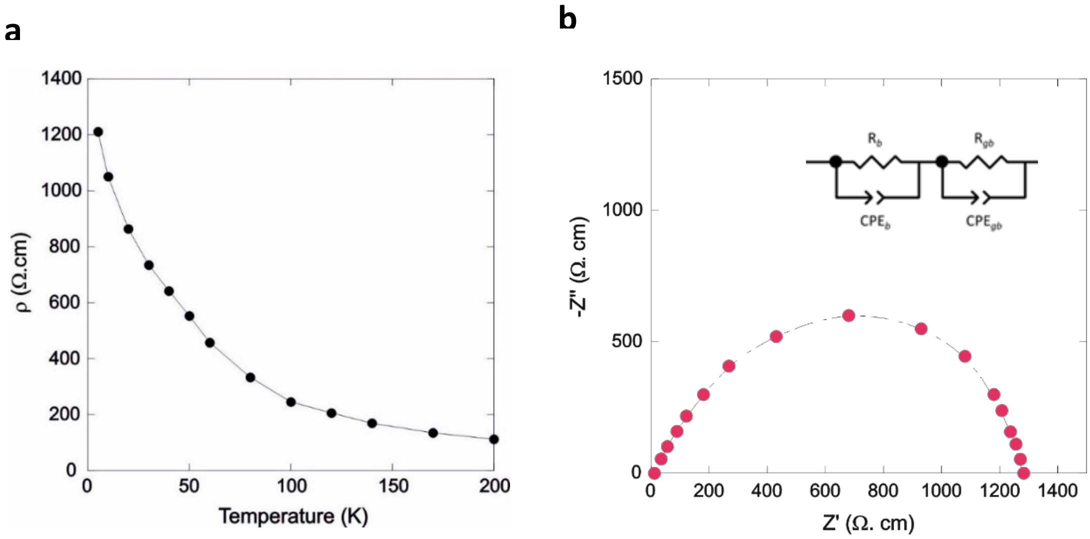

Supplementary Figure 10 (a) The variation of the grain boundary resistivity with temperature for $CeMnAsO_{0.95}F_{0.05}$. On cooling there is no evidence of T_{II} which is only observed in the bulk resistivity. **(b)** Complex impedance plots recorded at 10 K. The inset shows the equivalent circuit used to model the impedance data.

Impedance data were collected upon on cooling for $CeMnAsO_{0.95}F_{0.05}$. Impedance spectroscopy measurements were recorded with a Solartron 1260 impedance analyser in the frequency range 0.1 Hz – 1 MHz with an applied alternating voltage of 0.1 V. Impedance spectroscopy allows the separation of the grain boundary contribution to the total impedance of the material from the bulk contribution and demonstrate that the transition is not a result of a change in the resistivity of the grain boundary at T_{II}. The data were modelled by equivalent circuit fitting using the model shown in the inset to Supplementary Figure 10 (a) where R indicates a resistor, while CPE is a constant phase element; the subscript b stands for bulk, gb for grain boundary. The complex plane plot at 10 K is shown in Supplementary Figure 10(b).

Supplementary Note 3. Magnetic Properties of $CeMnAsO_{1-x}F_x$ and $Ce_{0.96}MnAsO_{1-x}F_x$

In CeMnAsO, the Mn^{2+} spins order along the *c* axis at T_{Mn} = 347 K with C-type long range antiferromagnetic ordering of the Ce moments and a spin reorientation of the Mn^{2+} spins at T_{SR} = 34 K [3-5]. Variable temperature magnetic susceptibility measurements evidence T_{SR} = 34 K for all samples of $CeMnAsO_{1-x}F_x$ (x = 0 – 0.075) (Supplementary Figures 11 and 12). There was no evidence of an anomaly at T_{II} for any of the samples in the variable temperature susceptibility data.

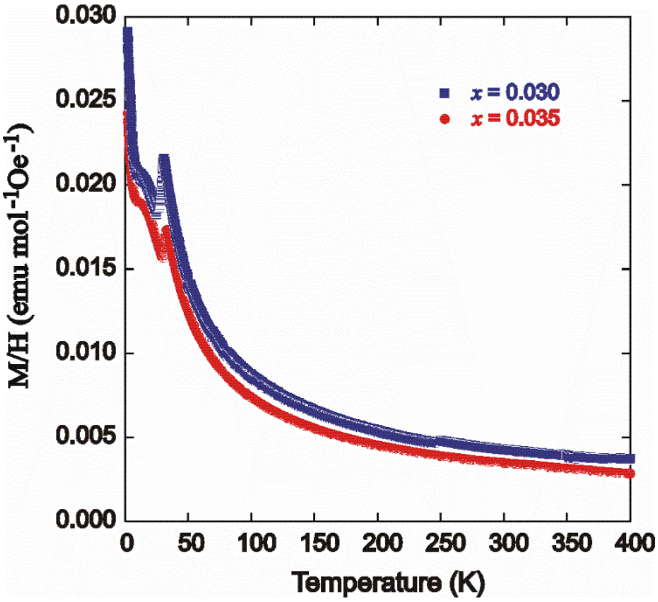

Supplementary Figure 11. The magnetic susceptibility of $CeMnAsO_{1-x}F_x$ (x = 0.030 and 0.035) showing the Ce ordering and Mn spin reorientation transition at T_{SR} = 34 K.

Powder neutron diffraction data were recorded for $Ce_{0.96}MnAsO_{0.95}F_{0.05}$ with T_{II} = 104 K. The Mn spins align along *c* below T_{Mn} ~ 350 K and the AFM ordering of Ce^{3+} and spin reorientation of the Mn moments is observed below T_{SR} = 34 K (Supplementary Figure 12(b)). There is no

change in the magnetic or nuclear structure at T_{II} and *P*4/*nmm* symmetry was retained down to 3.5 K (Supplementary Figures 12 and 13, Supplementary Tables 5 and 6).

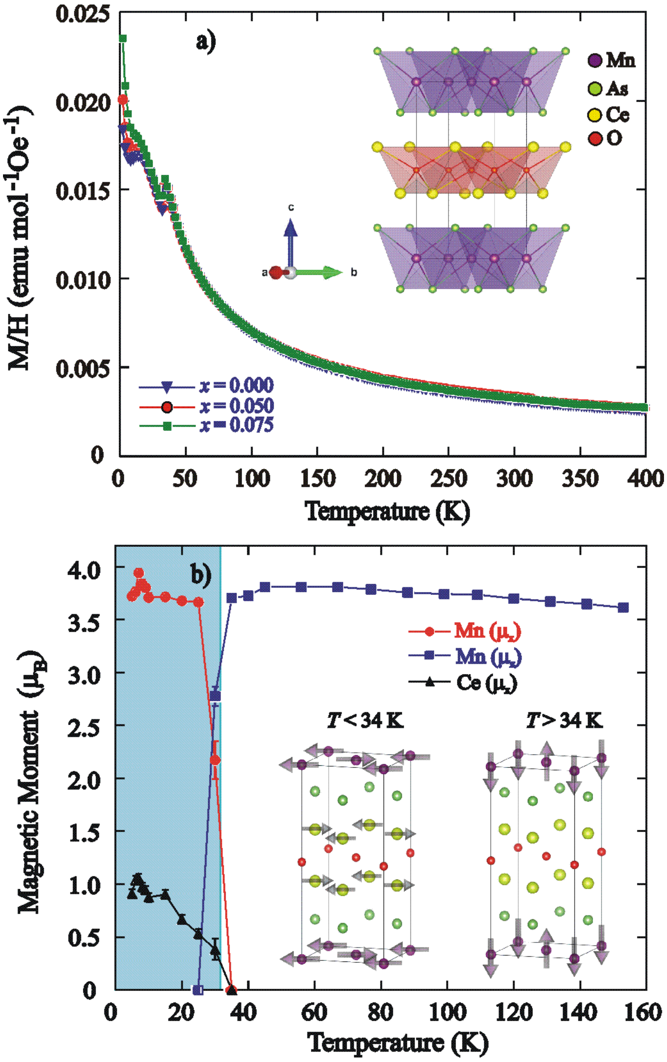

Supplementary Figure 12. Magnetic behaviour of $Ce_{0.96}MnAsO_{0.95}F_{0.05}$ and $CeMnAsO_{1-x}F_x$ (a) The magnetic susceptibility of $CeMnAsO_{1-x}F_x$ (x = 0, 0.050 and 0.075) showing the Ce ordering and Mn spin reorientation transition at T_{SR} = 34 K. The inset displays the tetragonal unit cell of CeMnAsO, which crystallises in the *P*4/*nmm* space group. Insulating CeO/F layers are situated between the tetrahedral As-Mn-As block. (b) The variation of Mn and Ce magnetic moments in $Ce_{0.96}MnAsO_{0.95}F_{0.05}$ with temperature. The inset shows the magnetic unit cells adopted above and below T_{SR}.

Supplementary Note 4. Temperature Dependence of the Crystal Structure

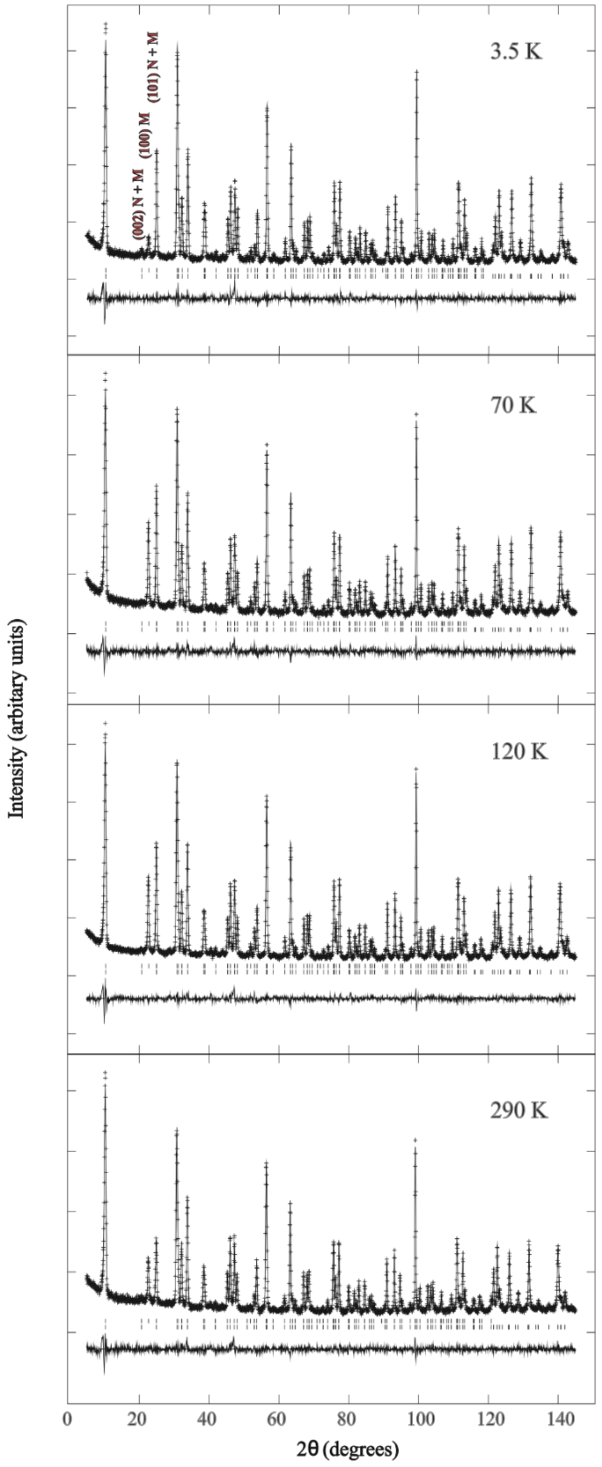

Supplementary Figure 13. Rietveld refinement fits to the *P*4/*nmm* structural model and magnetic structure described in Supplementary Figure 10 from neutron diffraction data of $Ce_{0.96}MnAsO_{0.95}F_{0.05}$ at selected temperatures between 290 K and 3.5 K. Top and bottom reflection positions represent the magnetic and nuclear structures respectively. N and M represent nuclear and magnetic diffraction respectively. The change in magnetic structure at T_{SR} is evidenced by the change in intensity of the (002), (100)/(010) and (101)/(011) magnetic diffraction peaks at $2\theta \sim 20.6°$, 22.6° and 24.9° respectively.

The high-resolution neutron powder diffraction patterns at selected temperatures are shown in Supplementary Figure 13. Supplementary Table 5 displays the refined values for the lattice parameters, atomic parameters and goodness of fit values as a function of temperature. The unit cell exhibits normal thermal expansion between 300 K – 40 K (Supplementary Figure S14(a)), with an anomaly in c at T_{SR}. The Mn-As and Ce-O bond lengths decrease upon cooling (Supplementary Figure 14(b)), while the As-Mn-As and Ce-O-Ce angles remain constant (Supplementary Table 6).

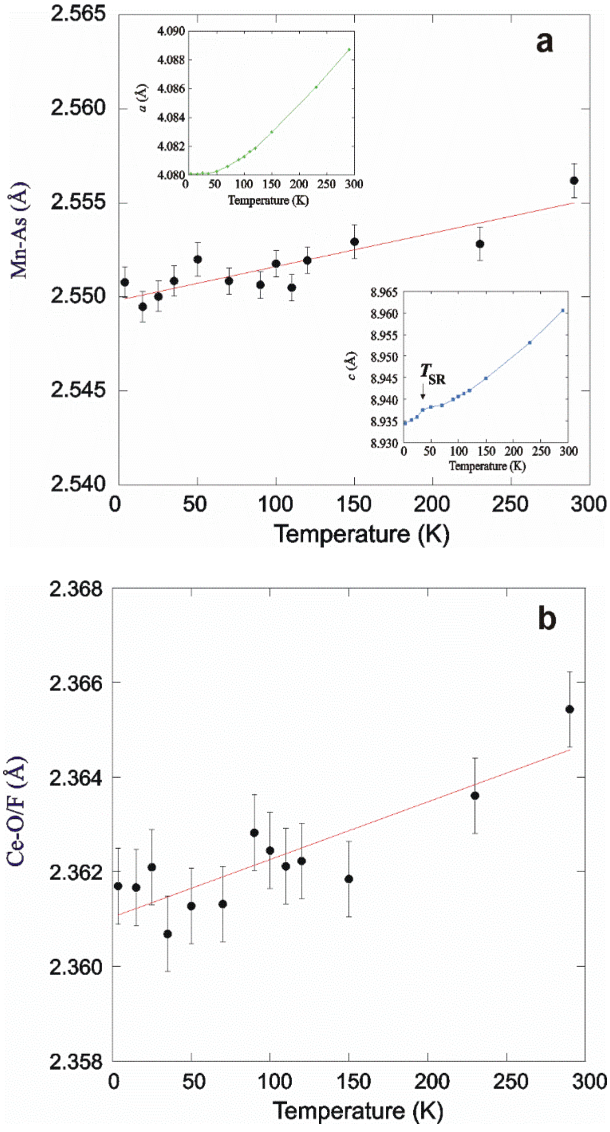

Supplementary Figure 14. The temperature dependence of (a) the tetrahedral Mn-As bond length and (b) the Ce-O/F bond length in $Ce_{0.96}MnAsO_{0.95}F_{0.05}$ determined from Rietveld fits against high resolution neutron diffraction data. The insets in (a) show the corresponding thermal variation of the lattice parameters, with a clear anomaly apparent in c at T_{SR} = 34 K. The error bars show the fitting errors from Rietveld refinement.

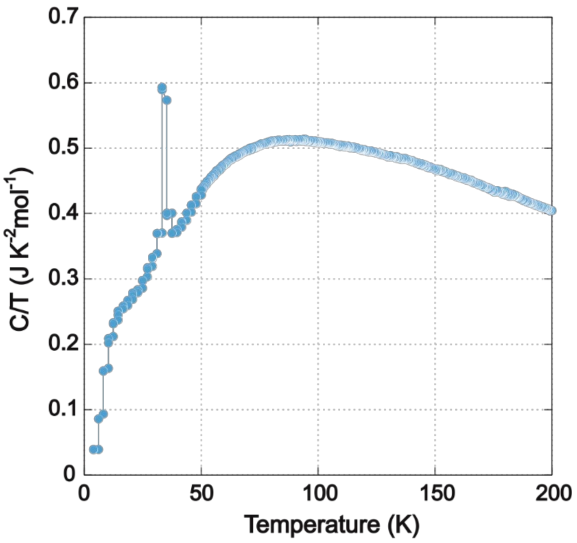

Supplementary Figure 15 Heat capacity of $Ce_{0.96}MnAsO_{0.95}F_{0.05}$ with T_{II} = 104 K. There is no evidence of a peak at T_{II} which suggests the transition is dynamic in nature. T_{SR} = 34 K is observed as a sharp peak in the data. The temperature variation of the heat capacity is very similar to that previously reported for CeMnAsO [3].

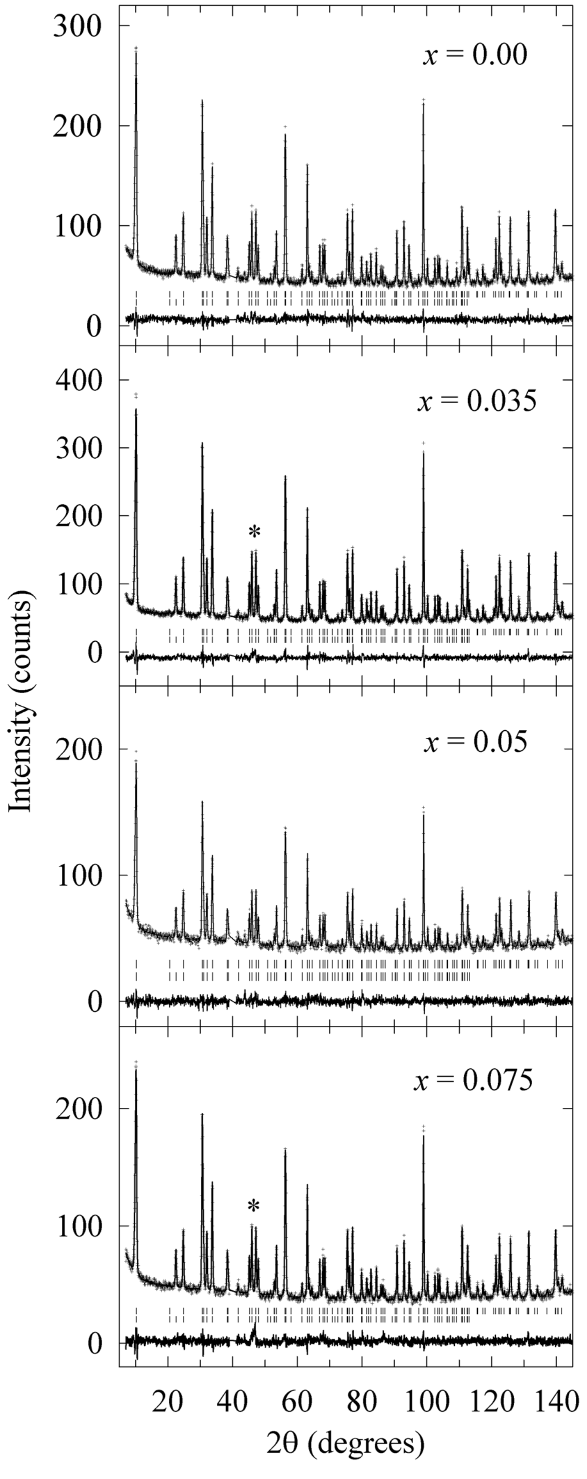

Supplementary Figure 16. Rietveld refinement fits to the *P*4/*nmm* structural model and magnetic structure described in Figure S11 from high resolution D2B neutron powder diffraction patterns at 300 K for $CeMnAsO_{1-x}F_x$. Top and bottom reflection positions represent the nuclear and magnetic structures respectively. The asterisks denote the positions of additional peaks from the sample environment.

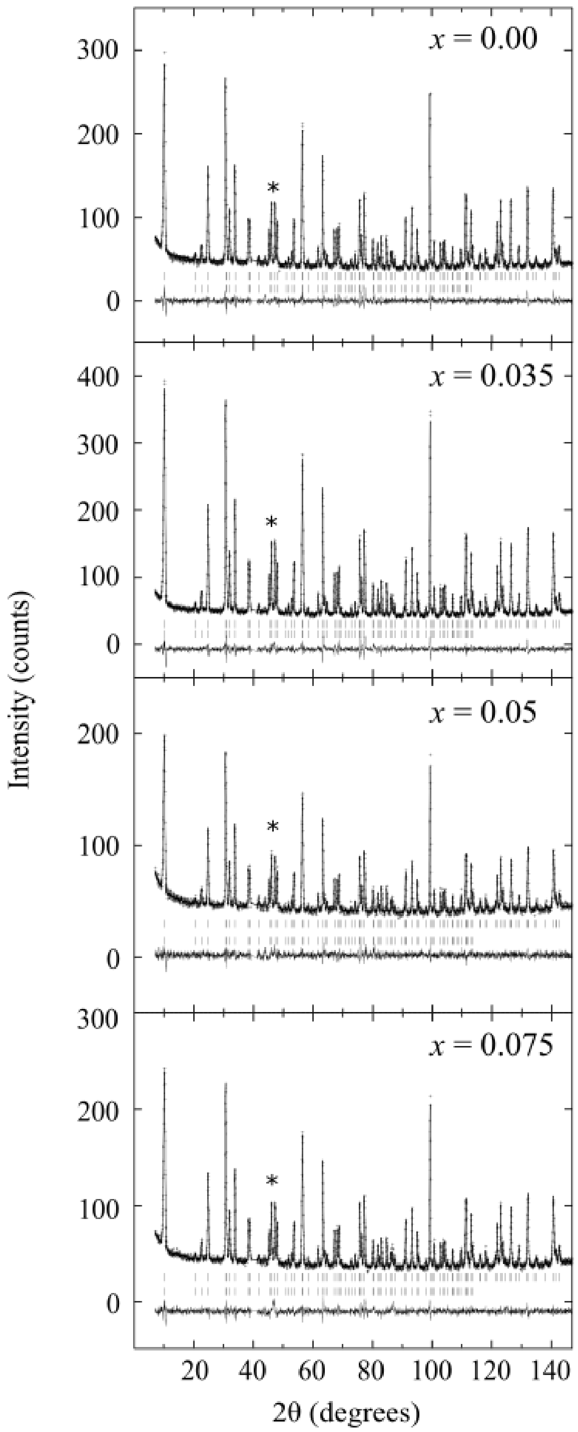

Supplementary Figure 17. Rietveld refinement fits to the *P*4/*nmm* structural model and magnetic structure described in Supplementary Figure 10 from high resolution D2B neutron powder diffraction patterns at ~ 10 K for $CeMnAsO_{1-x}F_x$. Top and bottom reflection positions represent the nuclear and magnetic structures respectively. The asterisks denote the positions of additional peaks from the sample environment. There is no change in crystal structure upon cooling from 300 K – 10 K.

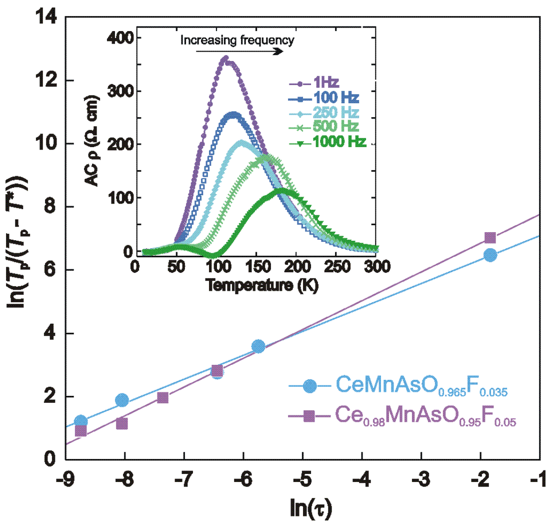

Supplementary Fig. 18 The AC transport properties of $Ce_{0.96}MnAsO_{0.95}F_{0.05}$ and $CeMnAsO_{0.965}F_{0.035}$. Temperature variation of the dissipative part of the AC resistivity of $Ce_{0.96}MnAsO_{0.95}F_{0.05}$ at selected frequencies (inset). The frequency dependence of the peak obeys the power law for $Ce_{0.96}MnAsO_{0.95}F_{0.05}$ and $CeMnAsO_{0.965}F_{0.035}$. The power law states: $\tau = \tau{*}\left[\frac{T_p}{(T_p - T{*})}\right]^{ZV}$ where τ is a characteristic time describing the dynamical fluctuation time scale, $\tau{*}$ is the relaxation time constant, T_p is the temperature of the peak maximum temperature at a given frequency, T^* is the critical temperature, z is the dynamical critical exponent and v is the correlation length exponent.

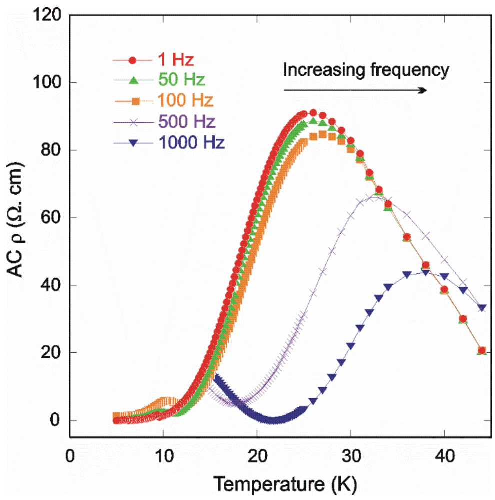

Supplementary Figure 19. Temperature variation of the dissipative part of the AC transport of $CeMnAsO_{0.0965}F_{0.035}$ at selected frequencies.

Supplementary Note 5. Density functional theory calculation details

The calculated band gap for CeMnAsO is 1.68 eV and this reduces to 1.04 eV for $CeMnAsO_{0.94}F_{0.06}$. A reduction in the band gap is also observed in the experimental data (Supplementary Table 4) as mid-gap states are created within the Mott-Hubbard gap. The calculated band gap is a sizable overestimate, which is likely to originate from the limitation of DFT to treat the strongly correlated nature of the quasiparticle band gap in this material.

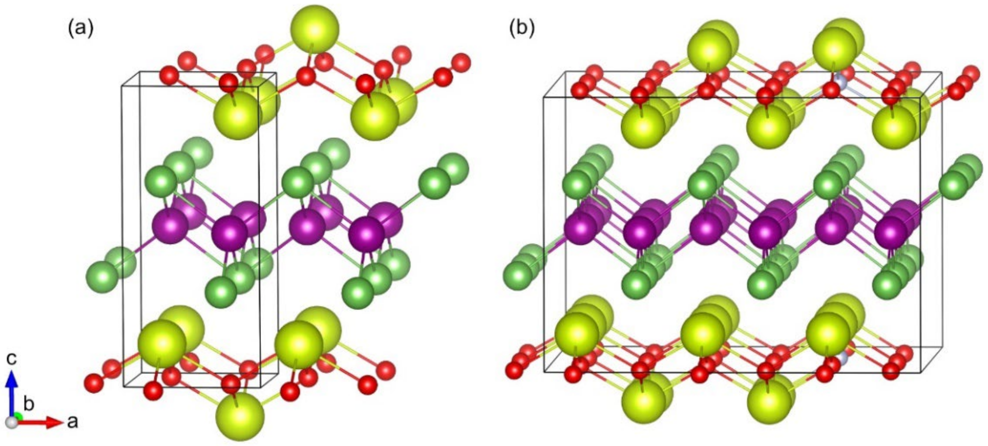

Supplementary Figure 20. The calculation cell for CeMnAsO and $CeMnAsO_{0.94}F_{0.06}$. a) CeMnAsO unit cell with two formula units. b) $CeMnAsO_{0.94}F_{0.06}$ supercell with the size of 3 × 3 × 1 unit cells. The yellow, purple, green and red spheres correspond to Ce, Mn, As and O, respectively.

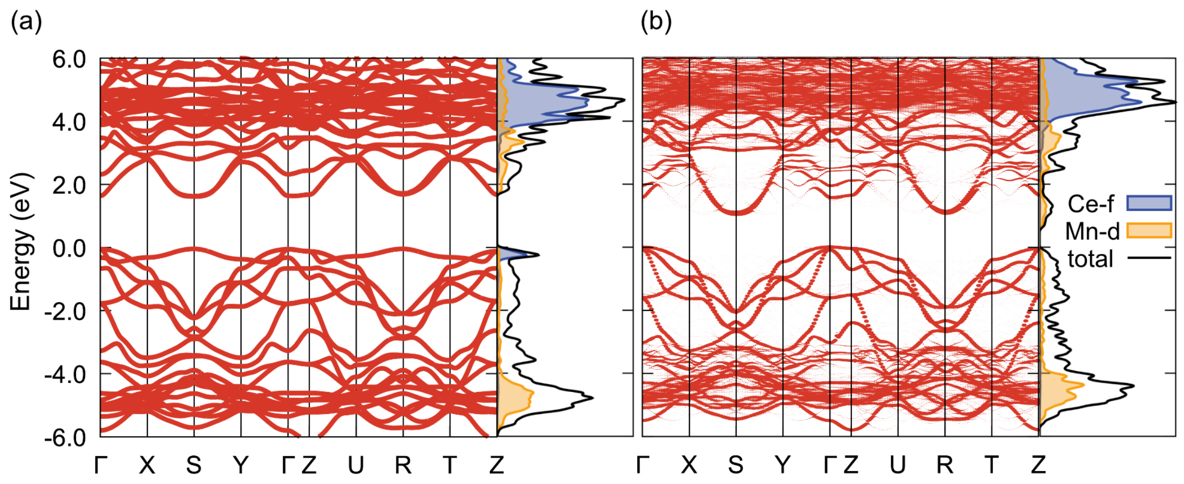

Supplementary Figure 21. Calculated electronic properties of CeMnAsO and $CeMnAsO_{0.94}F_{0.06}$. a) The electronic band structure and density of states of stoichiometric CeMnAsO. The partial density of states calculated with respect to the Ce f and Mn d projections are shaded in blue and yellow, respectively. The energy was shifted so that 0.0 eV corresponds to the Fermi energy. b) The unfolded electronic band structure and density of states of $CeMnAsO_{0.94}F_{0.06}$. The unfolding was done to match the reciprocal space path of the stoichiometric system. The energy was shifted so that 0.0 eV corresponds to the valence band maximum (Fermi energy at 2.0 eV). Both results are for the minority spin channel (the majority spin channel is shown in Figure 4).

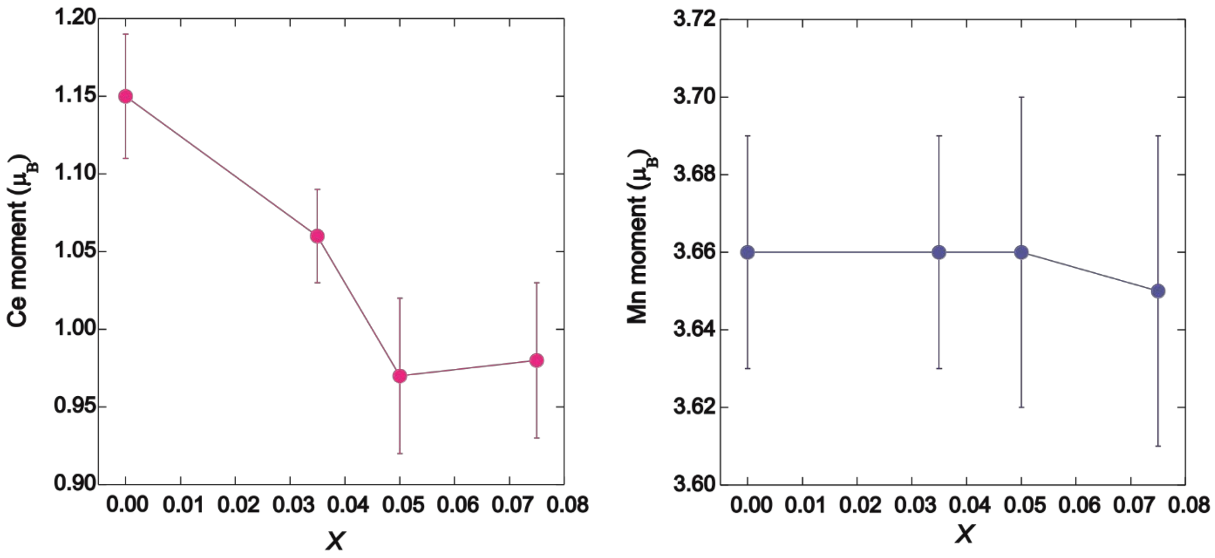

Supplementary Figure 22. Variation of the ordered (left) Ce and (right) Mn moments as obtained from neutron diffraction data recorded at 10 K on the high resolution D2B diffractometer at the ILL. The error bars show the fitting errors from Rietveld refinement.

Supplementary Table 5. Refined cell parameters, agreement factors and atomic parameters for $Ce_{0.96}MnAsO_{0.95}F_{0.05}$ from Rietveld fits against D2B neutron diffraction data at various temperatures. Ce and As are at 2c (¼, ¼, z), Mn at 2b (¾, ¼, ½) and O and F at 2a (¾ ,¼ ,0).

Atom	Occupancy		Temperature (K)						
			3.5	15	25	35	50	70	90
Ce	0.981(3)	*z*	0.1332(2)	0.1332(2)	0.1332(1)	0.1329(2)	0.1331(2)	0.1330(2)	0.1333(2)
		U_{iso} (Å^2)	0.0004(6)	0.0003(4)	0.0001(5)	0.0002(5)	0.0007(4)	0.0008(5)	0.0019(4)
Mn	1.00	U_{iso} (Å^2)	0.0003(4)	0.0002(4)	0.0004(4)	0.0002(4)	0.0010(4)	0.0006(3)	0.0012(3)
As	1.00	*z*	0.6713(2)	0.6712(2)	0.6712(1)	0.6714(2)	0.6715(2)	0.6713(2)	0.6712(1)
		U_{iso} (Å^2)	0.0005(3)	0.0005(3)	0.0003(3)	0.0006(2)	0.0013(2)	0.0006(3)	0.0014(2)
O/F	0.95/0.05	U_{iso} (Å^2)	0.0010(3)	0.0011(3)	0.0010(3)	0.0017(3)	0.0014(3)	0.0012(3)	0.0023(2)
		a (Å)	4.08012(4)	4.08006(4)	4.08013(5)	4.08010(4)	4.08027(4)	4.08061(4)	4.08108(3)
		c (Å)	8.9345(1)	8.9352(1)	8.9359(1)	8.9375(1)	8.9382(1)	8.9386(1)	8.9399(1)
		χ^2 (%)	1.998	2.056	1.977	2.046	2.031	2.024	2.489
		R_{WP} (%)	6.20	6.27	6.12	6.23	6.02	5.92	5.36
		R_P (%)	4.67	4.75	4.65	4.71	4.59	4.52	4.02

Atom	Occupancy		Temperature (K)					
			100	110	120	150	230	290
Ce	0.981(3)	z	0.1331(1)	0.1330(2)	0.1330(1)	0.1328(1)	0.1328(2)	0.1328(2)
		U_{iso} (Å^2)	0.0015(4)	0.0018(4)	0.0022(4)	0.0016(5)	0.0031(5)	0.0032(5)
Mn	1.00	U_{iso} (Å^2)	0.0014(4)	0.0012(3)	0.0017(3)	0.0016(4)	0.0031(4)	0.0046(4)
As	1.00	z	0.6714(1)	0.6711(1)	0.6713(1)	0.6713(1)	0.6710(2)	0.6712(1)
		U_{iso} (Å^2)	0.0022(2)	0.0020(2)	0.0022(2)	0.0028(3)	0.0045(3)	0.0054(3)
O/F	0.95/0.05	U_{iso} (Å^2)	0.0022(2)	0.0026(2)	0.0021(2)	0.0020(3)	0.0032(3)	0.0034(3)
		a (Å)	4.08128(4)	4.08164(3)	4.08187(3)	4.08298(4)	4.08610(4)	4.08872(4)
		c (Å)	8.9407(1)	8.9413(1)	8.9421(1)	8.9448(1)	8.9531(1)	8.9606(1)
		χ^2 (%)	2.541	2.419	2.452	2.036	1.990	1.827
		R_{WP} (%)	5.45	5.32	5.36	6.15	5.87	5.67
		R_P (%)	4.15	4.05	4.06	4.71	4.48	4.36

Supplementary Table 6: Selected bond lengths and angles for $Ce_{0.96}MnAsO_{0.95}F_{0.05}$ from Rietveld fits against D2B neutron diffraction data at various temperatures.

	Temperature (K)						
	3.5	15	25	35	50	70	90
**Bond Length (Å)**							
Ce-O/F	2.3617(8)	2.3617(8)	2.3621(8)	2.3607(8)	2.3615(8)	2.3613(8)	2.3628(7)
Mn-As	2.5507(8)	2.5496(8)	2.5502(8)	2.5510(8)	2.5518(8)	2.5508(9)	2.5506(7)
Mn-Mn	2.8851(3)	2.8850(3)	2.8851(3)	2.8851(3)	2.8852(3)	2.8854(3)	2.8857(1)
Ce-As	3.372(1)	3.374(1)	3.373(1)	3.374(1)	3.373(1)	3.374(1)	3.374(1)
**Bond Angle (^{o})**							
α_1 Ce-O/F-Ce	119.49(7)	119.49(7)	119.46(7)	119.58(7)	119.52(7)	119.55(7)	119.45(6)
α_2 Ce-O/F-Ce	104.71(3)	104.70(3)	104.72(3)	104.67(3)	104.69(3)	104.68(3)	104.73(3)
α_1 As-Mn-As	111.12(3)	111.09(3)	111.10(2)	111.13(3)	111.15(2)	111.12(3)	111.10(2)
α_2 As-Mn-As	106.23(5)	106.28(5)	106.26(5)	106.21(5)	106.16(5)	106.23(5)	106.26(4)

	**Temperature (K)**					
	100	110	120	150	230	290
**Bond Length (Å)**						
Ce-O/F	2.3625(7)	2.3621(7)	2.3622(7)	2.3619(8)	2.3636(9)	2.3655(9)
Mn-As	2.5518(7)	2.5506(7)	2.5520(7)	2.5527(9)	2.5528(9)	2.5561(9)
Mn-Mn	2.8859(1)	2.8862(2)	2.8863(2)	2.8871(3)	2.8893(3)	2.8912(3)
Ce-As	3.374(1)	3.376(1)	3.375(1)	3.377(1)	3.382(1)	3.383(1)
**Bond Angle (^{o})**						
α_1 Ce-O/F-Ce	119.49(6)	119.53(6)	119.54(6)	119.61(7)	119.62(7)	119.59(8)
α_2 Ce-O/F-Ce	104.71(3)	104.69(3)	104.69(3)	104.65(3)	104.65(3)	104.66(3)
α_1 As-Mn-As	111.13(2)	111.09(2)	111.13(2)	111.13(3)	111.07(3)	111.12(3)
α_2 As-Mn-As	106.20(4)	106.29(4)	106.21(4)	106.21(5)	106.32(5)	106.22(6)

References

1. Zhao, J., Huang, Q., De La Cruz, C., Li, S., Lynn, J. W., Chen, Y., Green, M. A., Chen, G. F., Li, G., Li, Z., Luo, J. L., Wang N. L. & Dai, P. Structural and magnetic phase diagram of $CeFeAsO_{1-x}F_x$ and its relation to high-temperature superconductivity. *Nat. Mater.* **7**, 953-959 (2008).

2. Markov, M., Rezaei, S. E., Sadeghi, S. N., Esfarjani, K. & Zebarjadi, M. Thermoelectric properties of semimetals. *Phys. Rev. Mater.* **3**, 095401 (2019).

3. Corkett, A. J., Free, D. G. & Clarke, S. J. Spin-reorientation transition in CeMnAsO. *Inorg. Chem.* **54**, 1178-1184 (2015).

4. Tsukamoto, Y., Okamoto, Y., Matsuhira, K., Whangbo, M. & Hiroi, Z. A magnetic transition probed by the Ce ion in square-lattice antiferromagnet CeMnAsO. *J. Phys. Soc. Jpn*. **80**, 094708 (2011).

5. Zhang, Q., Tian, W., Peterson, S. G., Dennis, K. W. & Vaknin, D. Spin reorientation and Ce-Mn coupling in antiferromagnetic oxypnictide CeMnAsO. *Phys. Rev. B*. **91**, 064418 (2015).
